# Factors predicting patient satisfaction in women with advanced breast cancer: a prospective study

**DOI:** 10.1186/s12885-018-4085-3

**Published:** 2018-02-07

**Authors:** Wendy W. T. Lam, Ava Kwong, Dacita Suen, Janice Tsang, Inda Soong, Tze Kok Yau, Winnie Yeo, Joyce Suen, Wing Ming Ho, Ka Yan Wong, Wing Kin Sze, Alice W. Y. Ng, Richard Fielding

**Affiliations:** 1 Centre for Psycho-Oncology Research & Training, Division of Behavioural Sciences, School of Public Health, The University of Hong Kong(HKU), 5/F, WMW Mong Block, Faculty of Medicine Building, 21 Sassoon Road, Pokulam, Hong Kong; 2 Department of Surgery, The University of Hong Kong, Pokfulam, Hong Kong; 3 Department of Clinical Oncology, The University of Hong Kong, Pokfulam, Hong Kong; 4 Department of Clinical Oncology, Pamela Youde Nethersole Eastern Hospital, Chai Wan, Hong Kong; 5 Department of Clinical Oncology, The Chinese University of Hong Kong, Sha Tin, Hong Kong; 6 Department of Oncology, Princess Margaret Hospital, Kwai Chung, Hong Kong; 7 Department of Clinical Oncology, Tuen Mun Hospital, Tuen Mun, Hong Kong

**Keywords:** Patient satisfaction, Advanced breast cancer, Chinese, Unmet information needs, Psychological distress

## Abstract

**Background:**

The present study (1) examined patient satisfaction with care over the first year following the diagnosis of advanced breast cancer and (2) tested if unmet health system and information needs, physical symptom distress, and psychological distress predicted patient satisfaction.

**Methods:**

Prospective study of 213 Chinese women with advanced breast cancer assessed while awaiting or receiving initial chemotherapy (baseline), then again at 1.5-, 3-, 6-, and 12-months post-baseline. Health system and information unmet (HSI) needs, psychological distress, physical symptom distress, and patient satisfaction were assessed at baseline; patient satisfaction was reassessed at each follow-up assessment. Latent growth curve analysis assessed changes in patient satisfaction over the 12 months follow-up; hierarchical multiple regression analysis tested if baseline health system information needs, physical symptom distress, anxiety and depression predicted patient satisfaction at one-year post-baseline.

**Results:**

The level of patient satisfaction was high and did not change significantly over time. Only HSI needs (β = − 0.27, *p* < 0.005) significantly associated with baseline patient satisfaction. Patient satisfaction at one-year post-baseline was predicted by HSI needs (β = − 0.26, *p* < 0.005), Anxiety (β = 0.23, *p* < 0.05) and Depression (β = − 0.28, *p* < 0.005), adjusting for the effect of baseline patient satisfaction (β = 0.22, *p* < 0.005).

**Conclusions:**

Unmet health information needs and greater depressive symptoms at initial treatment phased predicted subsequent poorer patient satisfaction. This highlights a need to reinforce the importance of patient-centered care model in managing advanced breast cancer.

## Background

Patient satisfaction is an important indicator of quality health care [1]. High patient satisfaction has been linked to better practice guideline adherence and lower inpatient mortality rates [2], as well as to greater patient acceptance and adherence to prescribed medical care [3, 4]. Patient satisfaction as an outcome measure is particularly important for patients diagnosed with a chronic or life-threatening condition, such as cancer, who require ongoing medical care to manage the condition. Numerous studies have examined patient satisfaction in oncology settings [5]. The level of patient satisfaction reported in these studies was generally high [5, 6]. Previous studies also examined factors associated with patient satisfaction including patient factors, such as age, gender, and type of cancer, and patient care factors, including information provision, doctor-patient relationship, and continuity of care. However, there is little consistency in reported associations between demographic factors and patient satisfaction [5]. In contrast, patient care factors, particularly information provision [5, 7], and a patient-centered consultation style [8–10] were important predictors of patient satisfaction. Furthermore, there is evidence that patient satisfaction might be hampered by unresolved physical symptom distress [11] and psychological distress [12]. While cancer patients are not one homogenous group, existing evidence for satisfaction is primarily based on studies of mixed cancer types. It is, therefore, important to examine patient satisfaction across types of cancer.

There is little information on patient satisfaction among women with advanced breast cancer. Advanced breast cancer includes both metastatic disease and locally advanced disease. Treatment advances have increased life expectancy for women with metastatic breast cancer, resulting a growing numbers of affected women living with the illness [13]. While treatment prolongs these women’s lives, disease progression is often inevitable and ongoing treatments impose various threats including side-effects, uncertainty, and fear of death. In contrast, locally-advanced breast cancer is a potentially-curable condition, but the prognosis is generally poor with a five-year survival of less than 50% [14]. Threats of cancer recurrence and aggressive cancer treatment side-effects put additional demands on affected women. Due to the nature of the disease, women with advanced breast cancer are likely to require ongoing cancer care. Hence, it is important to examine how such women assess their cancer care services. The current study aimed to examine patient satisfaction with care over the first year and its predictors in women following the diagnosis of advanced breast cancer, here, defined as metastatic breast cancer or regional disease spread. As previous studies have shown doctor-patient communication [5, 7] and patient-centered models of care [8–10] influenced patient satisfaction, we tested if patients’ disease and treatment information-related needs at baseline influenced subsequent patient satisfaction. We also tested if greater physical symptom distress [11] and psychological distress [12] at baseline predicted subsequent patient satisfaction.

## Methods

Following ethical committee approval for multi-center studies, Cantonese/Mandarin-speaking Chinese women newly diagnosed with stage III locoregional or stage IV metastatic breast cancer awaiting or receiving initial chemotherapy were recruited consecutively from six Hong Kong public oncology/breast cancer out-patient clinics between September 2008 and October 2012. Women with linguistic or intellectual difficulties were excluded from the study. At each hospital, potential patients were identified by clinical oncologists/surgeons. A trained research assistant then approached the potential patients immediately while they were awaiting their consultation. After explanations of the study, written consent was obtained from those who agreed to participate. Participants then completed a standardized baseline face-to-face questionnaire-based interview. Face-to-face follow-up assessments were then conducted at 6 weeks, 3-, 6-, and 12-months post-baseline at the oncology out-patient clinic.

### Measures

Patient satisfaction was assessed using the Nine-item Chinese Patient Satisfaction Questionnaire (ChPSQ-9) [15]. This measure was designed for assessing patient satisfaction with the specialist out-patient services provided by doctors, nurses, and other medical staff in the Chinese population [16]. The ChPSQ-9 primarily assessed the interpersonal, caring aspects of health care providers. It has been validated in the local cancer population including patients with breast cancer [15, 17]. Each item is rated on a 5-point Likert scale from “very dissatisfied” [1] to “very satisfied” [5]. Total scores range from 9 to 45, with higher scores indicating greater satisfaction. The ChPSQ-9 demonstrated good internal consistency, with Cronbach’s alpha ranged from 0.88 to 0.93.

The Chinese version of the Supportive Care Needs Survey Short Form (SCNS-SF 34-C) was used to assess type and magnitude of unmet needs [18–20]. The SCNS-SF 34 has been widely used and validated for use in cancer patients. This measure has good content validity and internal reliability (Cronbach’s alpha 0.82 to 0.92) [18, 19] and measures patients’ perceived need for help in five need domains: Health system and information (HSI) (11 items); Psychological (10 items); Physical and daily living (5 items); Sexuality (3 items); Patient care and support (5 items). Patients rate the intensity of each need over the past month for each item using five-point Likert scales: 1. No need: not applicable; 2. No need: satisfied; 3. Low need; 4. Moderate need; 5. High need. Scores were converted to standardized Likert summated scores ranging from 0 to 100 when calculating domain scores, with higher scores indicating greater perceived unmet need [20]. In the present study, the HSI subscale was used to assess patients’ disease and treatment information-related needs.

Psychological distress was measured by the 14-item Hospital Anxiety and Depression Scales (HADS) [21], comprised of 2 seven-item subscales that measure symptoms of anxiety and depression. Each item is rated on a four-point scale. Total scores for each subscale range from 0 to 21, with higher scores indicating greater distress. Scores exceeding 10 on each subscale constitute case definition for psychological morbidity, scores of 8–10 indicate subclinical caseness and scores < 8 represent non-cases. The Chinese version of the HADS has been widely used among cancer patients and has good validity [22]. Both anxiety (Cronbach’s α 0,86) and depression (Cronbach’s α 0.82) scales demonstrate good internal consistency. The HADS is suitable for use in cancer as it omits items addressing common vegetative symptom changes, such as fatigue or weight change, arising from physical illness which otherwise would inflate apparent depression and anxiety symptom prevalence in medical populations.

Physical symptom distress was measured using the Chinese version of the Memorial Symptom Assessment Scale Short-Form (MSAS-SF) a measure of 28 physical and 4 psychological symptoms commonly experienced by cancer patients [23, 24]. Patients indicate if they have experienced the listed symptoms over the past week and, if so, rate the intensity of distress (five-point Likert scale) from each symptom. The MSAS-SF consists of four subscales: a Global Distress Index, Physical symptom distress score, psychological symptom distress score, and the Total MSAS score [23, 24]. The MSAS-SF has been widely used and validated in cancer patients, the Chinese version having been validated in the local cancer population [23] the subscales demonstrating good internal consistency, with Cronbach’s alpha ranging from 0.84 to 0.91. In the present study, the Physical symptom distress subscale was used to assess physical symptom distress.

Patients’ socio-demographic data were collected at baseline interview, whereas clinical data were extracted from patients’ medical record using a standard protocol.

In the present study, all of the above measures were assessed at baseline, with patient satisfaction again measured at each subsequent follow-up assessment.

### Statistical analysis

Standard descriptive analyses assessed sample characteristics. We initially used latent growth curve (LGC) analysis for assessing the change of patient satisfaction over the 12 months’ follow-up. LGC analysis estimated the initial level of patient satisfaction and the associated slope, the rate of change in patient satisfaction scores. The analysis showed that level of patient satisfaction did not change significantly over time (data not shown). In this paper, we therefore reported the prediction of patient satisfaction at one-year post-baseline. Several variables, including health system and information needs and physical symptom distress consistently predict subsequent psychosocial distress [25, 26]. It seems likely that distressed patients would be less satisfied. Consequently, we used hierarchical multiple regression analysis to examine if baseline health system information needs, physical symptom distress, anxiety and depression also predicted patient satisfaction one-year post-baseline. The first block (model 1) entered in the hierarchical model consisted of significant demographic and clinical variables. In the second block (model 2), baseline patient satisfaction was entered. The third block (model 3) consisted of baseline health system information needs, physical symptom distress, anxiety and depression. All analyses reported in this paper were conducted using SPSS Statistics version 21.

## Results

**Fig. 1:**
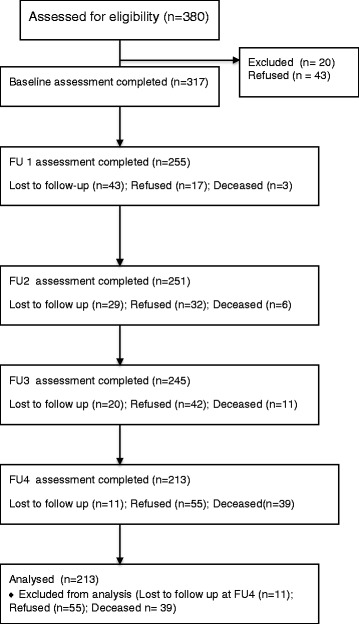
Sampling structure and attrition pattern of the study

Of 380 eligible women approached for recruitment, 20 were excluded due to linguistic or functional incapacity, and 317/360 (88%) gave informed consent and completed the interview (Fig. 1). With the exception of age, women refusing or lost to follow-up did not differ by demographic or medical factors, nor in their responses to the study variables at baseline. Women refusing or lost to follow-up were significantly older (mean 55.5 years old vs. 52 years old, t = − 2.28, *p* = 0.025) than those who completed the follow-up assessments. A total of 39 (11%) participants died during the study period. Compared to women who survived, those who died during the study were more likely to have recurrent breast cancer (71% vs. 40.9%, *p* = 0.002), have metastatic disease (71% vs. 50.2%, *p* = 0.03) and greater physical symptom distress scores (mean 1.08 vs. 0.56, t = 2.83, *p* = 0.008). For women who survived (*n* = 278) the duration of the study, 213 (77%) successfully completed the one-year post-baseline assessment. Hereafter, the descriptions of the baseline data and multivariate analysis examining predictors of patient satisfaction are based on data from these 213 women who completed the one-year post-baseline assessment. Table 1 summarizes the demographic and clinical characteristics of the study sample.

**Table 1:** Baseline demographic, clinical, and study variables measured for participants (*n* = 213)

Characteristics	Participants (%)
*Demographics*	
Age (years) mean ± standard deviation (SD)	52.03 ± 9.09
Marital status	
Married/cohabiting	144 (67.6)
Single/divorced/separated/widowed	69 (32.4)
Education level	
No/primary formal education	78 (36.6)
Secondary (completed high school)/Tertiary (college/university)	135 (63.3)
Total monthly household income (HK$)^a^	
< $10,000	74 (34.7)
$10,001–30,000	83 (39.0)
> $30,001	41 (19.3)
Missing	15 (7.0)
Occupation	
Full-time/part-time occupation	71 (33.3)
Retired	22 (10.3)
Housewife	68 (31.9)
Unemployed before/after diagnosis	50 (23.5)
Missing	2 (1.0)
*Clinical data*	
Recurrence of breast cancer	
Yes	77 (36.2)
No	136 (63.8)
Metastasis of breast cancer	
Yes	99 (46.5)
No	114 (53.5)
Time since current diagnosis (days)	
Mean ± SD (median)	19.86 ± 49.14 (8)
Type of surgery	
No surgery	68(31.9)
Breast conserving therapy	22 (10.3)
Modified radical mastectomy	113 (53.1)
Modified radical mastectomy plus reconstruction	10 (4.7)
Undergoing active treatment at baseline	
Chemotherapy	22 (10.3)
Radiation therapy	17 (7.9)
Hormonal therapy	8 (3.7)
Targeted therapy	10 (4.7)
*Study variables*	Mean (SD)
ChPSQ-9 baseline	34.97 (5.29)
ChPSQ-9 12-months post-baseline (*n* = 213)	35.30 (4.69)
SCNS HSI need domain baseline	35.43 (11.24)
MSAS Physical symptom distress subscale baseline	0.51 (0.37)
HADS Anxiety	3.95 (3.93)
HADS Depression	3.87 (3.86))

*SD* Standard deviation, *HK$* Hong Kong dollars ^a^1 US$ = 7.8 HK$

*ChPHQ-9* Chinese Patient Satisfaction Questionnaire

*SCNS HSI* Supportive care need scale– Health system information need

*MSAS* Memorial Symptom Assessment Scale

*HADS* Hospital Anxiety and Depression Scale

### Patient satisfaction, health system information needs, physical symptom and psychological distress

The mean total PSQ scale score was 34.97 (SD5.29) at baseline and 35.30 (SD4.69) at one-year post-baseline, suggesting the level of patient satisfaction was high and stable (Table 1). Repeated measure analysis showed no significant difference of patient satisfaction between baseline and follow-up assessments (F = 0.47, *p* = 049). The mean baseline SCNS Health system information needs score was 35.43 (SD 11.24), indicating a moderate level of unmet need regarding disease and treatment-related information and continuity of care. In contrast, the mean scores on the MSAS measure of physical symptom distress (mean 0.51, SD 0.37), HADS anxiety (mean 3.95, SD 3.93), and HADS depression (mean 3.87, SD 3.86) suggested low levels of physical symptom and psychological distress at baseline.

### Predictors of patient satisfaction

Linear regression analyses showed patient satisfaction at 12-months post-baseline was not significantly associated with patients’ demographic and clinical characteristics. Therefore, only baseline patient satisfaction (PSQ-9), patient disease and treatment-related health service and information needs (HSI needs), physical symptom distress (MSAS physical symptom distress), and psychological distress (HADS Anxiety and HADS Depression) were included in the subsequent hierarchical multiple regression analysis (Table 2). After adjusting for the effect of baseline PSQ-9, the inclusion of HSI needs increased the variance by an additional 5% (β = − 0.23, *p* < 0.005, model 2). Baseline MSAS physical symptom distress did not predict 12-months post-baseline PSQ scores (model 3). In the final model, the inclusion of HADS Anxiety (β = 0.23, *p* < 0.05, model 2) and Depression (β = − 0.28, *p* < 0.005) accounted only for an additional 3% of variance. Baseline PSQ-9 and HSI significantly predicted 12-month post-baseline PSQ. Adjusting for the effect of baseline PSQ-9, patients indicating higher baseline unmet disease and treatment-related needs and with higher baseline depression (HADS-D) scores reported significantly lower satisfaction at 1-year post-baseline, whereas patients with higher baseline anxiety (HADS-A) scores reported significantly greater 1-year post-baseline satisfaction. Post-hoc power analysis was conducted using G*Power 3.1. With an effect size of 0.14, a sample size of 213 and significance criteria of 0.05, the statistical power was 0.99, suggesting the sample size was sufficient to detect a difference.

**Table 2:** Hierarchical multiple regression models predicting patient satisfaction at one-year post-baseline by predictors

Baseline	Model 1		Model 2		Model 3		Model 4	
	β	95% CI	β	95% CI	β	95% CI	β	95% CI
PSQ scores	0.30**	0.15, 0.38	0.21**	0.06, 0.31	0.22**	0.07, 0.32	0.21**	0.06, 0.31
SCNS Health System & Information need scores			−0.23**	−0.16, − 0.04	−0.25**	− 0.17, − 0.05	−0.26**	− 0.18, − 0.06
MSAS physical symptom distress scores					0.07	−0.55, 1.84	0.13	−0.23, 2,56
HADS Anxiety scores							0.23*	0.04, 0.50
HADS Depression scores							−0.28**	−0.60, −0.09
R^2^	0.08		0.13		0.14		0.17	
R^2^ change	0.08		0.05		0.01		0.03	

*PSQ* Patient Satisfaction Questionnaire, *SCNS* Supportive Care Needs Scale, *MASA* Memorial Symptom Assessment Scale, *HADS* Hospital Anxiety and Depression Scale

*p < 0.05; **p < 0.005

Because baseline satisfaction scores were such strong predictors of subsequent satisfaction, we performed a post-hoc cross-sectional analysis to identify correlates of baseline PSQ-9 scores. Table 3 details factors associated with baseline PSQ-9. Baseline patient satisfaction did not associate with patients’ demographic or clinical characteristics in linear regression analyses. Therefore, only HSI needs, MSAS physical symptom distress, HADS Anxiety and HADS Depression were included in the post-hoc regression analysis. Only HSI need (β = − 0.27, *p* < 0.005) was significantly associated with baseline PSQ-9 scores. Patients with higher baseline unmet disease and treatment-related needs reported poorer patient satisfaction at baseline.

**Table 3:** Multiple regression model of patient satisfaction at baseline

	β	95% CI
SCNS Health System & Information need scores	−0.27**	−0.19, − 0.07
MSAS physical symptom distress scores	−0.05	−1.79, 0.92
HADS Anxiety scores	0.12	−0.21, 0.24
HADS Depression scores	−0.19	−0.47, 0.002
R^2^		0.38

*SCNS* Supportive Care Needs Scale, *MASA* Memorial Symptom Assessment Scale, *HADS* Hospital Anxiety and Depression Scale

*p < 0.05; **p < 0.005

## Discussion

The present study examined factors predicting patient satisfaction during the first year following the diagnosis of advanced breast cancer. Consistent with previous studies on patient satisfaction [5], our study showed most women diagnosed with advanced breast caner reported high level of satisfaction with their care over the duration of the study. The level of patient satisfaction was quite stable over time.

Overall, the study results provide partial support for the hypotheses. As hypothesized, greater perceived unmet needs for disease and treatment-related information and continuity of care during the initial treatment phase predicted subsequent poorer patient satisfaction. This concurred with previous studies that patient-centered models of care predict higher patient satisfaction [5, 7–10, 27, 28]. Our findings also supported the hypothesis that patients experiencing greater depressive symptoms were likely to be dissatisfied with medical care [12]. Depressed patients often hold negative views of themselves and the world and therefore may appear generally dissatisfied [12]. Also, depressed patients may be more dissatisfied as their psychosocial issues are not being addressed during consultation [12]. There is evidence that oncologists often fail to provide much psychosocial support during consultations [9, 29].

Contrary to our hypothesis, women with more anxiety symptoms reported greater subsequent patient satisfaction. It may be that anxious patients were more likely to raise their concerns with the oncologists, get answers and therefore feel more satisfied with their medical care. Despite efforts to the contrary, many oncologists seldom actively explore and identify patients’ concerns, but will usually appropriately respond to concerns raised by patients [9]. Physical symptom distress did not predict patient satisfaction. Previous studies showed patient dissatisfaction to be related to inadequate information provision on managing physical symptoms, but not the frequency of physical symptoms [11]. The results of the post-hoc analysis we performed are consistent with this. Satisfaction at baseline was inversely associated only with HIS needs. These findings reiterate the importance of addressing patients’ information and psychosocial needs as much as physical needs. Lastly, our findings indicated that patient demographic factors had little effect on patient satisfaction. There is little consistent evidence that patient demographic characteristics influence patient satisfaction [5].

This study has several limitations. First, we used a generic measure of patient satisfaction, but one designed for use in the specialist out-patient services that these patients attended. The measure assesses care provided by doctors and also nurses, and emphasizes care-related communications [15]. It may not capture all the issues in relation to oncology care, such as more technical information. Future studies should consider using a more oncology-specific patient satisfaction measure such as Patient Satisfaction and Quality in Oncological Care questionnaire [30]. Second, the baseline was assessed only at the start of chemotherapy and therefore was unable to examine prior patient satisfaction at the diagnostic phase.

## Conclusions

We understand the present study to be the first longitudinal study describing patient satisfaction among women diagnosed with advanced breast cancer. The findings highlight most of this sample of Chinese women with advanced breast cancer were satisfied with their medical care. However, high level of unmet health system and information needs predicted longer-term poor patient satisfaction. This highlights the need to reinforce the importance of patient-centered care model in consultations for managing advanced breast cancer.

## Abbreviations

ChPSQ-9: The Nine-item Chinese Patient Satisfaction Questionnaire
SCNS-SF 34-C: The Chinese version of the Supportive Care Needs Survey Short Form
HSI: Health System Information
HADS: Hospital Anxiety and Depression Scales
MSAS-SF: Memorial Symptom Assessment Scale Short-Form
LGC: Latent Growth Curve

## References

[1] Jackson JL, Chamberlin J, Kroenke K (2001). Predictors of patient satisfaction. Soc Sci Med.

[2] Glickman SW, Boulding W, Manary M, Staelin R, Roe MT, Wolosin RJ (2010). Patient satisfaction and its relationship with clinical quality and inpatient mortality in acute myocardial infarction. Circ Cardiovasc Qual Outcomes.

[3] Borras JM, Sanchez-Hernandez A, Navarro M, Martinez M, Mendez E, Ponton JL (2001). Compliance, satisfaction, and quality of life of patients with colorectal cancer receiving home chemotherapy or outpatient treatment: a randomised controlled trial. BMJ.

[4] Kahn KL, Schneider EC, Malin JL, Adams JL, Epstein AM (2007). Patient centered experiences in breast cancer: predicting long-term adherence to tamoxifen use. Med Care.

[5] Lis CG, Rodeghier M, Gupta D (2009). Distribution and determinants of patient satisfaction in oncology: a review of the literature. Patient Prefer Adherence.

[6] Lis CG, Rodeghier M, Grutsch JF, Gupta D (2009). Distribution and determinants of patient satisfaction in oncology with a focus on health related quality of life. BMC Health Serv Res.

[7] Ong LM, Visser MR, Lammes FB, de Haes JC (2000). Doctor-patient communication and cancer patients' quality of life and satisfaction. Patient Educ Couns.

[8] Brown RF, Hill C, Burant CJ, Siminoff LA (2009). Satisfaction of early breast cancer patients with discussions during initial oncology consultations with a medical oncologist. Psychooncology.

[9] Lam WW, Kwok M, Chan M, Hung WK, Ying M, Or A (2014). Does the use of shared decision-making consultation behaviors increase treatment decision-making satisfaction among chinese women facing decision for breast cancer surgery?. Patient Educ Couns.

[10] Walker MS, Ristvedt SL, Haughey BH (2003). Patient care in multidisciplinary cancer clinics: does attention to psychosocial needs predict patient satisfaction?. Psychooncology.

[11] Feyer P, Kleeberg UR, Steingräber M, Günther W, Behrens M (2008). Frequency of side effects in outpatient cancer care and their influence on patient satisfaction--a prospective survey using the PASQOC questionnaire. Support Care Cancer.

[12] Bui QU, Ostir GV, Kuo YF, Freeman J, Goodwin JS (2005). Relationship of depression to patient satisfaction: findings from the barriers to breast cancer study. Breast Cancer Res Treat.

[13] Stebbing J, Ngan S (2010). Breast cancer (metastatic). BMJ Clin Evid.

[14] Lee MC, Newman LA (2007). Management of patients with locally advanced breast cancer. Surg Clin N Am.

[15] Wong WS, Fielding R, Wong CM, Hedley AJ (2008). Psychometric properties of the nine-item chinese patient satisfaction questionnaire (chpsq-9) in chinese patients with hepatocellular carcinoma. Psychooncology.

[16] Bulter LD, Hedley AJ, Cheang J (1995). Quality from the patient’s perspective: developing an instrument to measure patient satisfaction with specialist outpatient service of the Hospital Authority. Final report to the Hospital Authority.

[17] Wong WS, Fielding R, Wong C, Hedley A (2009). Confirmatory factor analysis and sample invariance of the chinese patient satisfaction questionnaire (chpsq-9) among patients with breast and lung cancer. Value Health.

[18] Au A, Lam WW, Kwong A, Suen D, Tsang J, Yeo W (2011). Validation of the chinese version of the short-form supportive care needs survey questionnaire (SCNS-SF34-C). Psychooncology.

[19] Li WW, Lam WW, Shun SC, Lai YH, Law WL, Poon J, Fielding R (2013). Psychometric assessment of the chinese version of the supportive care needs survey short-form (SCNS-SF34-C) among hong kong and taiwanese chinese colorectal cancer patients. PLoS One.

[20] McElduff P, Boyes A, Zucca A, Girgis A (2004). The supportive care needs survey: a guide to administration, scoring and analysis.

[21] Zigmond AS, Snaith RP (1983). The hospital anxiety and depression scale. Acta Psychiatr Scand.

[22] Leung CM, Wing YK, Kwong PK, Lo A, Shum K (1999). Validation of the chinese-cantonese version of the hospital anxiety and depression scale and comparison with the hamilton rating scale of depression. Acta Psychiatr Scand.

[23] Lam WW, Law CC, Fu YT, Wong KH, Chang VT, Fielding R (2008). New insights in symptom assessment: the chinese versions of the memorial symptom assessment scale short form (MSAS-SF) and the condensed MSAS (CMSAS). J Pain Symptom Manag.

[24] Chang VT, Hwang SS, Kasimis B, Thaler HT (2004). Shorter symptom assessment instruments: the condensed memorial symptom assessment scale (CMSAS). Cancer Investig.

[25] Lam WW, Bonanno GA, Mancini AD, Ho S, Chan M, Hung WK (2010). Trajectories of psychological distress among chinese women diagnosed with breast cancer. Psychooncology.

[26] Lam WW, Soong I, Yau TK, Wong KY, Tsang J, Yeo W (2013). The evolution of psychological distress trajectories in women diagnosed with advanced breast cancer: a longitudinal study. Psychooncology.

[27] Sanders SL, Bantum EO, Owen JE, Thornton AA, Stanton AL (2010). Supportive care needs in patients with lung cancer. Psychooncology.

[28] Chen JY, Tao ML, Tisnado D, Malin J, Ko C, Timmer M (2008). Impact of physician-patient discussions on patient satisfaction. Med Care.

[29] Hack TF, Pickles T, Ruether JD, Weir L, Bultz BD, Degner LF (2010). Behind closed doors: systematic analysis of breast cancer consultation communication and predictors of satisfaction with communication. Psychooncology.

[30] Kleeberg UR, Feyer P, Günther W, Behrens M (2008). Patient satisfaction in outpatient cancer care: a prospective survey using the PASQOC questionnaire. Support Care Cancer.

